# Synthesis and structure of 1′,4′-diphenyl-1a,1′,4′,4′′,5′′,9b-hexa­hydro-2′′*H*-di­spiro­[cyclopropa[*l*]phenanthrene-1,2′-[1,4]ep­oxy­naphthalene-3′,3′′-thio­phene]

**DOI:** 10.1107/S2056989025004104

**Published:** 2025-05-13

**Authors:** Yuewei Wen, Dasan M. Thamattoor

**Affiliations:** a Department of Chemistry Colby College,Waterville ME 04901 USA; University of Aberdeen, United Kingdom

**Keywords:** crystal structure, polycyclic compound, cyclo­addition, Hirshfeld analysis

## Abstract

The title compound, C_39_H_30_OS, was inadvertently prepared as a Diels–Alder adduct between 1,3-di­phenyl­isobenzo­furan and 3-(1a,9b-di­hydro-1*H*-cyclo­propa[*l*]phenanthren-1-yl­idene)tetra­hydro­thio­phene. A combination of fused, bridged, and spiro­cyclic ring systems are all featured within a single mol­ecular structure of this highly crowded polycyclic compound.

## Abstract 2

The title compound was inadvertently prepared as a Diels–Alder adduct between 1,3-di­phenyl­isobenzo­furan and 3-(1a,9 b-di­hydro-1*H*-cyclo­propa[*l*]phenanthren-1-yl­idene)tetra­hydro­thio­phene. A combination of fused, bridged, and spiro­cyclic ring systems are all featured within a single mol­ecular structure of this highly crowded polycyclic compound.

## 1. Chemical context

We recently reported (see Scheme below) that the photolysis of 3-(1a,9b-di­hydro-1*H*-cyclo­propa[*l*]phenanthren-1-yl­idene)tetra­hydro­thio­phene (**1**) produces the alkyl­idenecarbene **2** with the concomitant loss of phenanthrene (Anderson *et al.*, 2023 ▸). Carbene **2** subsequently rearranges into the strained thia­cyclo­hexyne **3**. When the photolysis was carried out in the presence of 1,3-di­phenyl­isobenzo­furan (**4**), in an attempt to inter­cept **3**, the title compound **5**, which is the Diels–Alder adduct between **1** and **4**, was obtained instead. The crystal structure and Hirshfeld surface analysis of **5** is described herein.
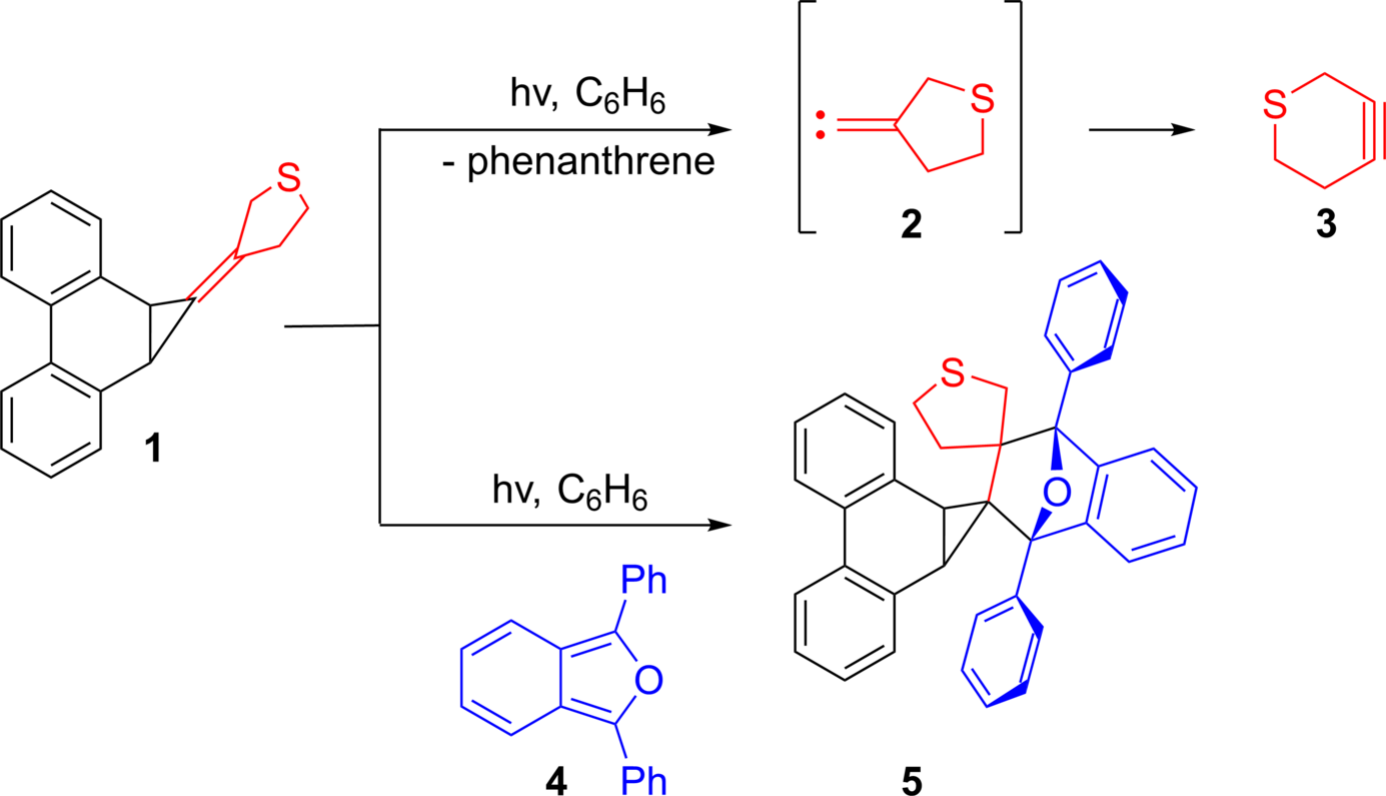


## 2. Structural commentary

**Figure 1:**
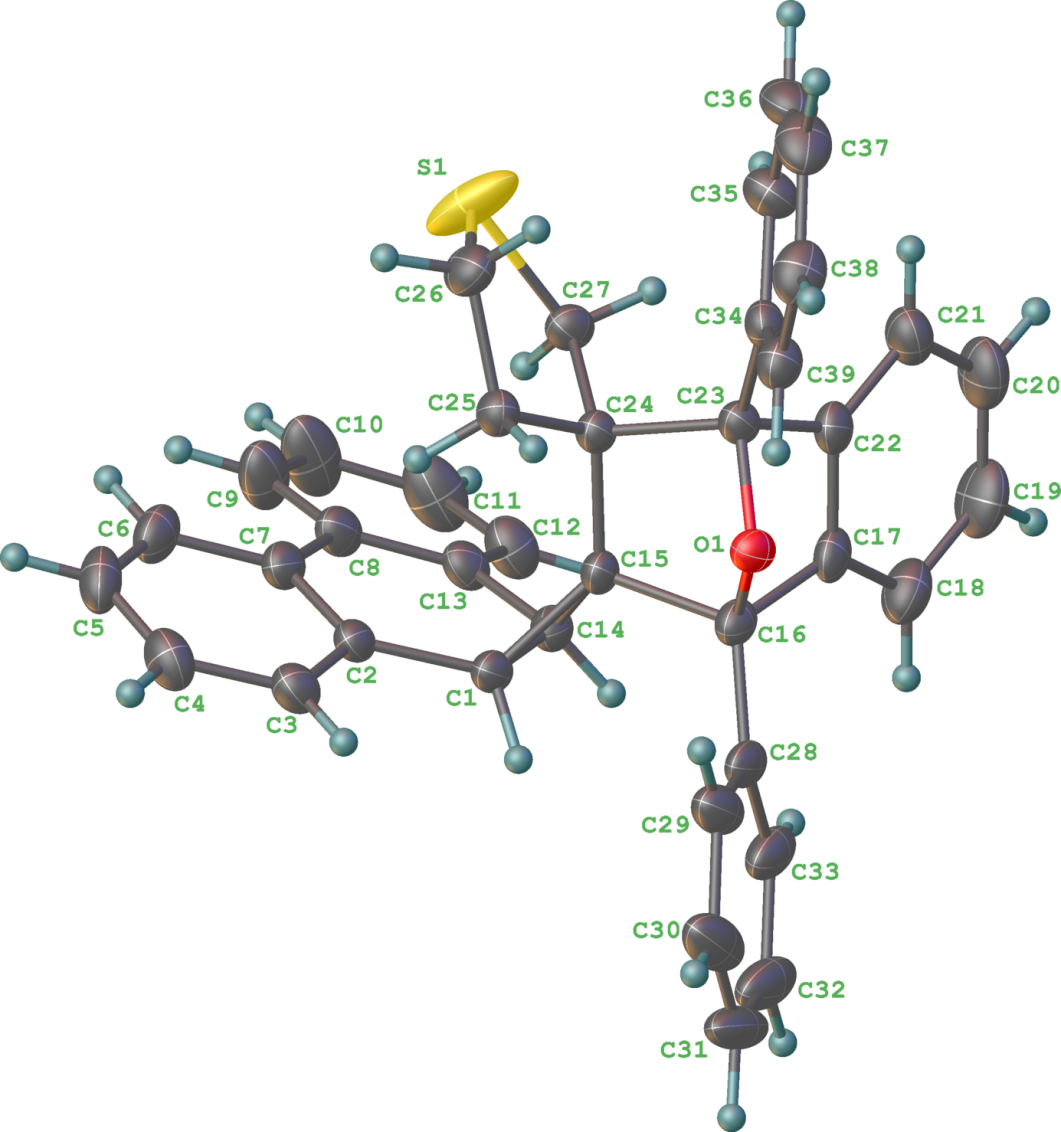
The mol­ecular structure of **5**. Displacement ellipsoids are shown at the 50% probability level.

The mol­ecular structure of **5**, C_39_H_30_OS, is shown in Fig. 1 ▸. The structure shows that the thia­cyclo­pentyl ring is on the same side of the mol­ecule as the dibenzonorcarane framework, which is understandable as **4** likely prefers to approach from the less sterically encumbered *exo* side of **1** to form the 4 + 2 cyclo­adduct.

The highly crowded adduct **5** displays a variety of ring connections within a single mol­ecule. Besides the fused rings that were inherited from the dibenzonorcarane moiety in **1**, the structure of **5** also includes the bridged bicyclic system, 7-oxabi­cyclo­[2.2.1]heptane, which is involved in spiro­cyclic connections with the thia­cyclo­pentyl and cyclo­propyl rings on one side and a fused connection to a benzene ring on the other side. There are six stereogenic centres (C1, C14, C15, C16, C23, C24) in **5**; in the arbitrarily chosen asymmetric unit, they have *R*, *R*, *R*, *R*, *S* and *S* configurations, respectively, but crystal symmetry generates a racemic mixture. Both the C16/C17/C22/C23/O1 and C16/C15/C24/C23/O1 five-membered rings are well described as envelopes with the shared O atom as the flap in both cases, whereas the C24/C25/C26/S1/C27 ring is twisted on the C25—C26 bond. The dihedral angles between the C17–C22 phenyl group and the pendant C28–C33 and C34–C39 rings are 28.56 (10) and 47.14 (9)°, respectively; the dihedral angle between the pendant rings is 21.53 (10)°. Some of the C—C—C bond angles associated with the fused rings are notably distorted, such as C17—C16—C28 = 120.88 (15)° and C16—C17—C22 = 104.55 (15)°. The centroid of the thia­cyclo­pentane ring is 3.5062 (11) Å from the centroid of the six-membered ring of the norcarane group. Ten intra­molecular short contacts between atoms (shorter than sum of vdW radii – 0.3 Å) were identified and are presented in Table 1 ▸.

## 3. Supra­molecular features

**Figure 2:**
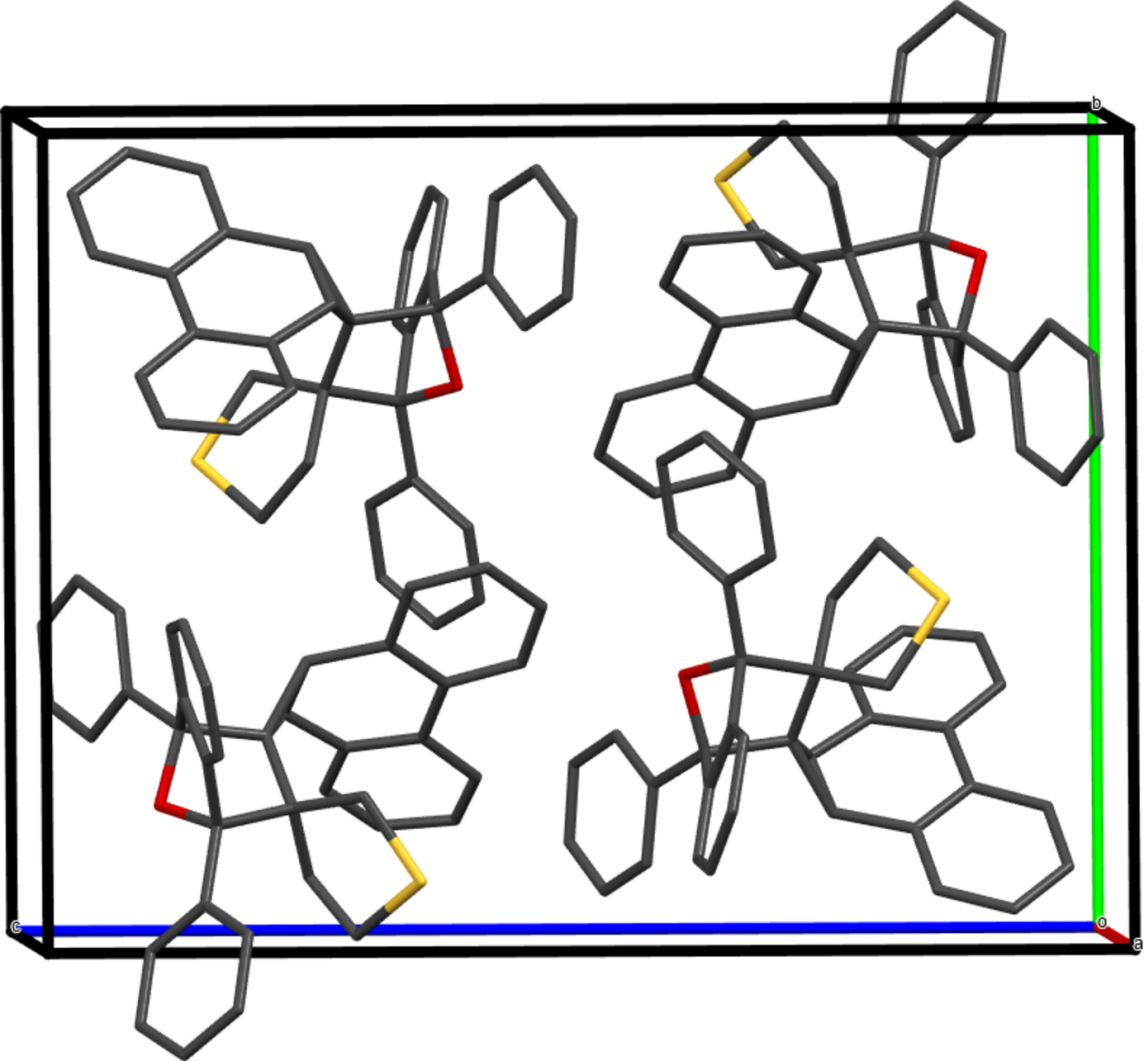
The monoclinic unit cell of **5** with four mol­ecules.

**Figure 3:**
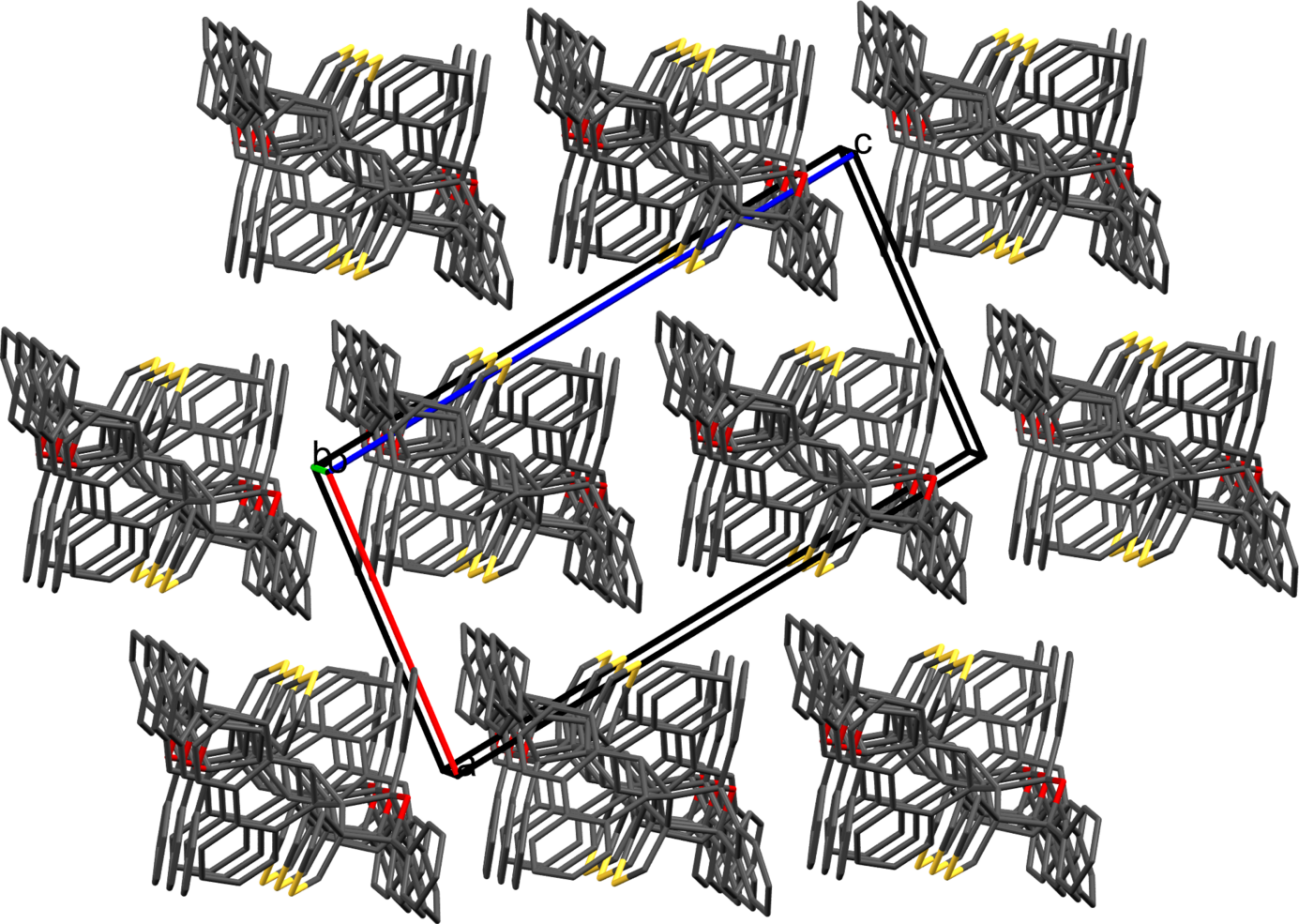
Packing diagram of of **5** viewed along the *b-*axis direction.

The unit cell of **5**, comprising four mol­ecules, is shown in Fig. 2 ▸, and a section of the packing diagram viewed along the *b-*axis direction is depicted in Fig. 3 ▸. The only identified directional contact in the extended structure of **5** is a weak C31—H31⋯S1^i^ [symmetry code: (i) −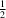 + *x*, 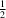 − *y*, 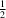 + *z*] hydrogen bond with H⋯S = 2.85 Å and C—H⋯S = 135°, which generates [001] chains.

**Figure 4:**
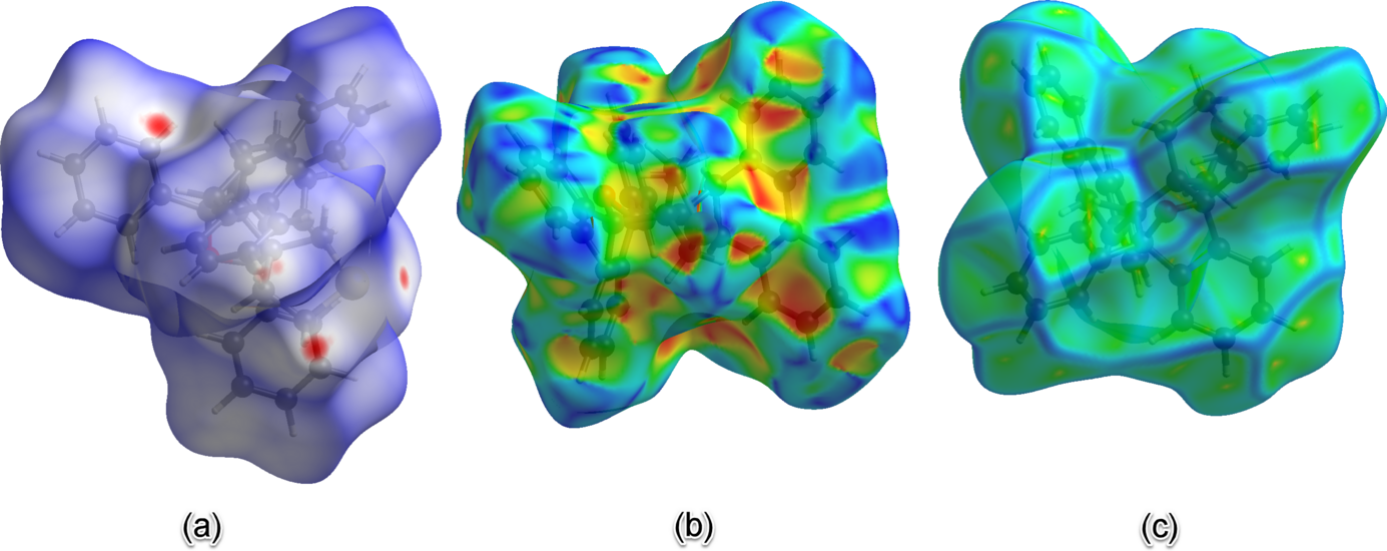
The Hirshfeld surface shown as: (*a*) *d*_norm_ plot over the range −0.12 to 1.43 Å, (*b*) shape index plot over the range −1.00 to 1.00 Å, and (*c*) curvedness map plot over the range −4.00 to 4.00 Å.

A *d*_norm_ Hirshfeld surface (Spackman & Jayatilaka, 2009 ▸) was generated for **5** with *Crystal Explorer 21.5* (Spackman *et al.*, 2021 ▸) to investigate inter­molecular contacts, and is shown in Fig. 4 ▸*a*. There are very few significant short contacts among mol­ecules of **5** as can be seen from the limited number of red regions, which are mostly quite small, on the surface. These contacts primarily stem from the thia­cyclpentane ring and one of the phenyl rings attached to the bridgehead of the 7-oxabi­cyclo­[2.2.1]heptane group. The shape-index map (Fig. 4 ▸*b*) reveals hardly any π–π inter­actions but complementary red and blue regions indicate possible C—H⋯π inter­actions. The curvedness map (Fig. 4 ▸*c*) shows a ‘crumpled’ structure with many valleys and ridges but no extensive planarity that could facilitate π–π inter­actions.

**Figure 5:**
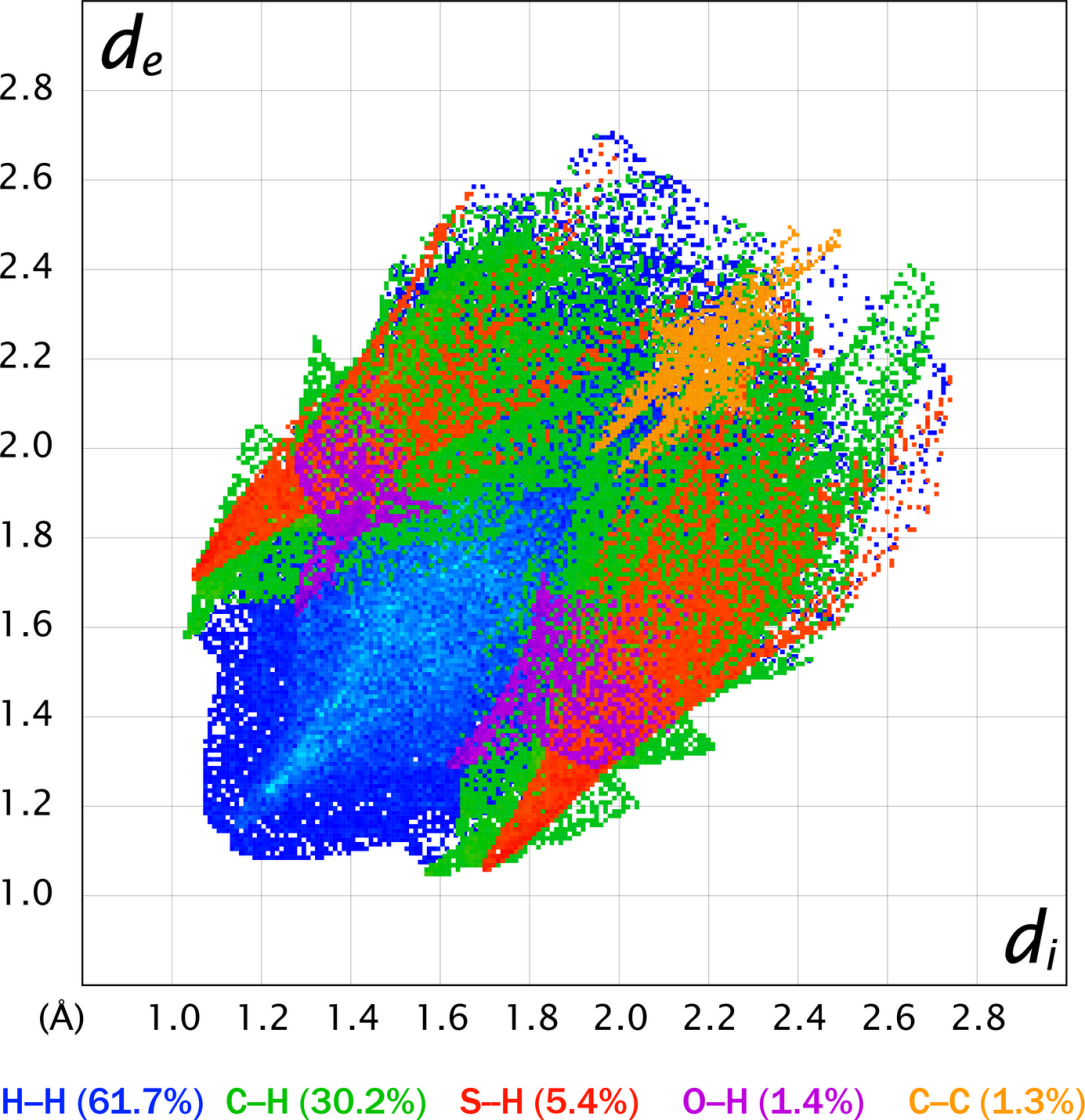
The reciprocal two-dimensional fingerprint plot of *d*_e_*versus d*_i_ showing the different types of inter­molecular contacts by color.

A composite, color-coded reciprocal two-dimensional fingerprint plot (Spackman & McKinnon, 2002 ▸) of *d*_i_*versus d*_e_, where *d*_i_ and *d*_e_ are distances from any given point on the surface to the nearest inter­nal and external atom, respectively, is shown in Fig. 5 ▸. This plot indicates that H⋯H (61.7%) and C⋯H/H⋯C (30.2%) inter­actions account for nearly 92% of the inter­molecular contacts within the crystal structure of **5**. The remaining contributions come from S⋯H/H⋯S (5.4%), O⋯H/H⋯O (1.4%), and C⋯C (1.3%) contacts.

## 4. Database survey

A survey of the Cambridge Structural Database (CSD; Groom *et al.*, 2016 ▸) using WebCSD (version 1.9.66; accessed April 29, 2025) did not find the title compound **5** reported previously. The survey also showed that there were 23 structures featuring the dibenzonorcarane unit. Of these, only three (CSD refcodes DOTJOP, DOTJUV, Roth *et al.*, 2024 ▸; HOJLIF, Roth & Thamattoor, 2024 ▸) had the cyclo­propyl ring in a spiro­cyclic system.

## 5. Synthesis and crystallization

The title compound **5** was inadvertently synthesized while performing the photolysis of **1** (556 mg, 2 mmol) with **4** (559 mg, 2 mmol) in benzene (5 ml). It was isolated in extremely low yield (0.6%) after flash chromatography on silica gel using hexa­nes as the eluent. Crystals suitable for X-ray diffraction were obtained by slow evaporation of column fractions.

## 6. Refinement

Crystal data, data collection and structure refinement details are summarized in Table 2 ▸. H atoms were positioned geometrically (C—H = 0.95–0.99 Å) and refined as riding with *U*_iso_(H) = 1.2*U*_eq_(C).

## Supplementary Material

Crystal structure: contains datablock(s) I. DOI: 10.1107/S2056989025004104/hb8140sup1.cif

Structure factors: contains datablock(s) I. DOI: 10.1107/S2056989025004104/hb8140Isup2.hkl

CCDC reference: 2444424

Additional supporting information:  crystallographic information; 3D view; checkCIF report

## Figures and Tables

**Table 1:** Intra­molecular short contacts (Å) in **5**

Atom1⋯Atom2	Length	Atom1⋯Atom2	Length
O1⋯H29	2.38	O1⋯H39	2.33
C2⋯H25*A*	2.35	C13⋯H27*A*	2.38
C22⋯H27*B*	2.50	C28⋯H1	2.40
C34⋯H26*A*	2.58	C35⋯H26*A*	2.52
H6⋯H9	2.07	H18⋯H33	2.05

**Table 2:** Experimental details

Crystal data	
Chemical formula	C_39_H_30_OS
*M*_r_	546.69
Crystal system, space group	Monoclinic, *P*2_1_/*n*
Temperature (K)	173
*a*, *b*, *c* (Å)	10.2352 (14), 14.3939 (14), 19.120 (3)
β (°)	98.121 (12)
*V* (Å^3^)	2788.6 (6)
*Z*	4
Radiation type	Mo *K*α
μ (mm^−1^)	0.15
Crystal size (mm)	0.21 × 0.15 × 0.10

Data collection	
Diffractometer	Bruker D8 QUEST ECO
Absorption correction	Multi-scan (*SADABS*; Krause *et al.*, 2015 ▸)
*T*_min_, *T*_max_	0.700, 0.745
No. of measured, independent and observed [*I* > 2σ(*I*)] reflections	34580, 5692, 4424
*R*_int_	0.035
(sin θ/λ)_max_ (Å^−1^)	0.625

Refinement	
*R*[*F*^2^ > 2σ(*F*^2^)], *wR*(*F*^2^), *S*	0.048, 0.117, 1.04
No. of reflections	5692
No. of parameters	370
H-atom treatment	H-atom parameters constrained
Δρ_max_, Δρ_min_ (e Å^−3^)	0.51, −0.71

Computer programs: *APEX4* and *SAINT-Plus* V8.40B (Bruker, 2021 ▸), *SHELXT* (Sheldrick, 2015*a* ▸), *SHELXL* (Sheldrick, 2015*b* ▸) and *OLEX2* (Dolomanov *et al.*, 2009 ▸).

## supplementary crystallographic information

### 1',4'-Diphenyl-1a,1',4',4'',5'',9b-hexahydro-2''H-dispiro[cyclopropa[l]phenanthrene-1,2'-[1,4]epoxynaphthalene-3',3''-thiophene] Crystal data

**Table:**

C_39_H_30_OS	*F*(000) = 1152
*M**_r_* = 546.69	*D*_x_ = 1.302 Mg m^−^^3^
Monoclinic, *P*2_1_/*n*	Mo *K*α radiation, λ = 0.71073 Å
*a* = 10.2352 (14) Å	Cell parameters from 9835 reflections
*b* = 14.3939 (14) Å	θ = 2.6–26.4°
*c* = 19.120 (3) Å	µ = 0.15 mm^−^^1^
β = 98.121 (12)°	*T* = 173 K
*V* = 2788.6 (6) Å^3^	Prism, clear colourless
*Z* = 4	0.21 × 0.15 × 0.10 mm

### 1',4'-Diphenyl-1a,1',4',4'',5'',9b-hexahydro-2''H-dispiro[cyclopropa[l]phenanthrene-1,2'-[1,4]epoxynaphthalene-3',3''-thiophene] Data collection

**Table:**

Bruker D8 QUEST ECO diffractometer	5692 independent reflections
Radiation source: sealed X-ray tube, Siemens KFFMO2K–90C	4424 reflections with *I* > 2σ(*I*)
Curved graphite monochromator	*R*_int_ = 0.035
Detector resolution: 7.9 pixels mm^-1^	θ_max_ = 26.4°, θ_min_ = 2.8°
ω and φ scans	*h* = −12→11
Absorption correction: multi-scan (SADABS; Krause *et al.*, 2015)	*k* = −18→17
*T*_min_ = 0.700, *T*_max_ = 0.745	*l* = −23→23
34580 measured reflections	

### 1',4'-Diphenyl-1a,1',4',4'',5'',9b-hexahydro-2''H-dispiro[cyclopropa[l]phenanthrene-1,2'-[1,4]epoxynaphthalene-3',3''-thiophene] Refinement

**Table:**

Refinement on *F*^2^	Primary atom site location: dual
Least-squares matrix: full	Hydrogen site location: inferred from neighbouring sites
*R*[*F*^2^ > 2σ(*F*^2^)] = 0.048	H-atom parameters constrained
*wR*(*F*^2^) = 0.117	*w* = 1/[σ^2^(*F*_o_^2^) + (0.0449*P*)^2^ + 1.7735*P*] where *P* = (*F*_o_^2^ + 2*F*_c_^2^)/3
*S* = 1.04	(Δ/σ)_max_ < 0.001
5692 reflections	Δρ_max_ = 0.51 e Å^−^^3^
370 parameters	Δρ_min_ = −0.71 e Å^−^^3^
0 restraints	

### 1',4'-Diphenyl-1a,1',4',4'',5'',9b-hexahydro-2''H-dispiro[cyclopropa[l]phenanthrene-1,2'-[1,4]epoxynaphthalene-3',3''-thiophene] Special details

**Table:**

Geometry. All esds (except the esd in the dihedral angle between two l.s. planes) are estimated using the full covariance matrix. The cell esds are taken into account individually in the estimation of esds in distances, angles and torsion angles; correlations between esds in cell parameters are only used when they are defined by crystal symmetry. An approximate (isotropic) treatment of cell esds is used for estimating esds involving l.s. planes.

### 1',4'-Diphenyl-1a,1',4',4'',5'',9b-hexahydro-2''H-dispiro[cyclopropa[l]phenanthrene-1,2'-[1,4]epoxynaphthalene-3',3''-thiophene] Fractional atomic coordinates and isotropic or equivalent isotropic displacement parameters (Å^2^)

**Table:**

	*x*	*y*	*z*	*U*_iso_*/*U*_eq_	
S1	0.50970 (8)	0.41369 (5)	0.15667 (4)	0.0686 (2)	
O1	0.44774 (11)	0.32159 (8)	0.39494 (6)	0.0243 (3)	
C1	0.21870 (16)	0.21536 (12)	0.27250 (8)	0.0242 (4)	
H1	0.172212	0.194188	0.312277	0.029*	
C2	0.13212 (16)	0.25996 (12)	0.21334 (9)	0.0252 (4)	
C3	0.02796 (18)	0.31451 (13)	0.22878 (10)	0.0323 (4)	
H3	0.012505	0.319988	0.276449	0.039*	
C4	−0.05379 (19)	0.36102 (14)	0.17650 (11)	0.0380 (5)	
H4	−0.125098	0.397612	0.187890	0.046*	
C5	−0.0298 (2)	0.35332 (14)	0.10711 (11)	0.0398 (5)	
H5	−0.083540	0.386277	0.070746	0.048*	
C6	0.0713 (2)	0.29818 (14)	0.09073 (10)	0.0362 (5)	
H6	0.086403	0.293921	0.042933	0.043*	
C7	0.15291 (18)	0.24800 (13)	0.14271 (9)	0.0288 (4)	
C8	0.24977 (18)	0.17904 (14)	0.12482 (9)	0.0313 (4)	
C9	0.2579 (2)	0.15425 (18)	0.05462 (10)	0.0469 (6)	
H9	0.206655	0.187384	0.017452	0.056*	
C10	0.3381 (2)	0.0833 (2)	0.03820 (12)	0.0576 (7)	
H10	0.342044	0.068378	−0.009858	0.069*	
C11	0.4130 (2)	0.03367 (18)	0.09116 (12)	0.0529 (6)	
H11	0.467554	−0.015965	0.079862	0.064*	
C12	0.4078 (2)	0.05710 (15)	0.16109 (11)	0.0389 (5)	
H12	0.458939	0.022875	0.197677	0.047*	
C13	0.32889 (17)	0.12991 (13)	0.17858 (9)	0.0286 (4)	
C14	0.32479 (17)	0.14966 (12)	0.25452 (9)	0.0261 (4)	
H14	0.335878	0.093116	0.285180	0.031*	
C15	0.36395 (16)	0.23926 (12)	0.29390 (8)	0.0226 (4)	
C16	0.41919 (17)	0.22644 (12)	0.37444 (8)	0.0258 (4)	
C17	0.55821 (18)	0.18749 (12)	0.37587 (9)	0.0281 (4)	
C18	0.6166 (2)	0.10124 (14)	0.38863 (10)	0.0412 (5)	
H18	0.567022	0.049790	0.401533	0.049*	
C19	0.7495 (2)	0.09164 (16)	0.38213 (12)	0.0504 (6)	
H19	0.790663	0.032781	0.390675	0.060*	
C20	0.8226 (2)	0.16548 (16)	0.36359 (11)	0.0469 (5)	
H20	0.913322	0.157079	0.359511	0.056*	
C21	0.76479 (19)	0.25235 (15)	0.35078 (10)	0.0357 (4)	
H21	0.814975	0.303723	0.338266	0.043*	
C22	0.63206 (17)	0.26214 (12)	0.35671 (9)	0.0263 (4)	
C23	0.53653 (16)	0.34333 (12)	0.34482 (8)	0.0229 (4)	
C24	0.44049 (16)	0.32564 (11)	0.27199 (8)	0.0223 (3)	
C25	0.35150 (17)	0.41160 (12)	0.25493 (9)	0.0258 (4)	
H25A	0.266860	0.392578	0.226991	0.031*	
H25B	0.332092	0.440339	0.299371	0.031*	
C26	0.42069 (19)	0.48164 (13)	0.21327 (10)	0.0342 (4)	
H26A	0.482016	0.520723	0.245453	0.041*	
H26B	0.355581	0.522429	0.184948	0.041*	
C27	0.51737 (18)	0.30983 (13)	0.20922 (9)	0.0278 (4)	
H27A	0.478300	0.257214	0.180133	0.033*	
H27B	0.610486	0.294498	0.226875	0.033*	
C28	0.32541 (19)	0.18490 (14)	0.41952 (9)	0.0328 (4)	
C29	0.24205 (19)	0.24318 (17)	0.45050 (10)	0.0416 (5)	
H29	0.250992	0.308669	0.447239	0.050*	
C30	0.1447 (2)	0.2052 (2)	0.48655 (11)	0.0581 (7)	
H30	0.087799	0.244976	0.508024	0.070*	
C31	0.1315 (3)	0.1098 (2)	0.49090 (11)	0.0645 (8)	
H31	0.064266	0.084173	0.514600	0.077*	
C32	0.2143 (3)	0.0523 (2)	0.46137 (11)	0.0597 (8)	
H32	0.205633	−0.013121	0.465296	0.072*	
C33	0.3107 (2)	0.08884 (16)	0.42580 (10)	0.0445 (5)	
H33	0.367904	0.048205	0.405312	0.053*	
C34	0.59274 (17)	0.43911 (12)	0.35865 (9)	0.0251 (4)	
C35	0.69184 (18)	0.47270 (14)	0.32219 (10)	0.0337 (4)	
H35	0.727457	0.433680	0.289538	0.040*	
C36	0.7387 (2)	0.56281 (14)	0.33335 (12)	0.0412 (5)	
H36	0.804790	0.585589	0.307545	0.049*	
C37	0.6896 (2)	0.61945 (14)	0.38189 (11)	0.0423 (5)	
H37	0.721962	0.680984	0.389631	0.051*	
C38	0.5933 (2)	0.58619 (13)	0.41900 (10)	0.0387 (5)	
H38	0.560281	0.624748	0.452835	0.046*	
C39	0.54436 (18)	0.49685 (13)	0.40730 (9)	0.0294 (4)	
H39	0.477228	0.474942	0.432741	0.035*	

### 1',4'-Diphenyl-1a,1',4',4'',5'',9b-hexahydro-2''H-dispiro[cyclopropa[l]phenanthrene-1,2'-[1,4]epoxynaphthalene-3',3''-thiophene] Atomic displacement parameters (Å^2^)

**Table:**

	*U*^11^	*U*^22^	*U*^33^	*U*^12^	*U*^13^	*U*^23^
S1	0.1024 (6)	0.0599 (4)	0.0558 (4)	0.0274 (4)	0.0535 (4)	0.0297 (3)
O1	0.0262 (6)	0.0279 (6)	0.0188 (6)	−0.0041 (5)	0.0036 (5)	−0.0013 (5)
C1	0.0240 (9)	0.0310 (9)	0.0173 (8)	−0.0051 (7)	0.0017 (6)	0.0003 (7)
C2	0.0212 (9)	0.0314 (9)	0.0222 (8)	−0.0060 (7)	−0.0002 (7)	0.0008 (7)
C3	0.0264 (10)	0.0399 (11)	0.0299 (9)	−0.0049 (8)	0.0017 (7)	−0.0017 (8)
C4	0.0286 (10)	0.0380 (11)	0.0448 (11)	0.0031 (8)	−0.0034 (8)	−0.0025 (9)
C5	0.0429 (12)	0.0343 (11)	0.0363 (11)	0.0006 (9)	−0.0148 (9)	0.0028 (8)
C6	0.0458 (12)	0.0374 (11)	0.0224 (9)	−0.0049 (9)	−0.0052 (8)	0.0014 (8)
C7	0.0276 (9)	0.0350 (10)	0.0222 (8)	−0.0067 (8)	−0.0016 (7)	−0.0003 (7)
C8	0.0281 (10)	0.0431 (11)	0.0219 (9)	−0.0064 (8)	0.0013 (7)	−0.0042 (8)
C9	0.0367 (12)	0.0783 (17)	0.0249 (10)	0.0049 (11)	0.0016 (8)	−0.0067 (10)
C10	0.0424 (13)	0.099 (2)	0.0315 (11)	0.0078 (13)	0.0049 (10)	−0.0235 (12)
C11	0.0388 (12)	0.0702 (16)	0.0497 (13)	0.0075 (11)	0.0061 (10)	−0.0269 (12)
C12	0.0314 (10)	0.0435 (12)	0.0402 (11)	0.0000 (9)	0.0002 (8)	−0.0095 (9)
C13	0.0249 (9)	0.0335 (10)	0.0265 (9)	−0.0062 (8)	0.0005 (7)	−0.0062 (7)
C14	0.0265 (9)	0.0279 (9)	0.0225 (8)	−0.0042 (7)	−0.0010 (7)	0.0011 (7)
C15	0.0226 (9)	0.0279 (9)	0.0164 (7)	−0.0009 (7)	0.0003 (6)	0.0014 (6)
C16	0.0301 (9)	0.0269 (9)	0.0189 (8)	−0.0043 (7)	−0.0013 (7)	0.0012 (7)
C17	0.0329 (10)	0.0303 (9)	0.0181 (8)	0.0000 (8)	−0.0063 (7)	0.0002 (7)
C18	0.0516 (13)	0.0327 (11)	0.0358 (11)	0.0027 (10)	−0.0065 (9)	0.0055 (8)
C19	0.0548 (14)	0.0439 (13)	0.0493 (13)	0.0233 (11)	−0.0041 (11)	0.0067 (10)
C20	0.0349 (12)	0.0567 (14)	0.0470 (12)	0.0172 (10)	−0.0018 (9)	0.0027 (11)
C21	0.0278 (10)	0.0439 (12)	0.0341 (10)	0.0043 (9)	0.0003 (8)	0.0000 (9)
C22	0.0271 (9)	0.0298 (9)	0.0200 (8)	0.0011 (7)	−0.0034 (7)	−0.0014 (7)
C23	0.0218 (8)	0.0277 (9)	0.0192 (8)	−0.0012 (7)	0.0027 (6)	−0.0006 (7)
C24	0.0216 (8)	0.0262 (9)	0.0188 (8)	−0.0010 (7)	0.0020 (6)	0.0002 (6)
C25	0.0241 (9)	0.0277 (9)	0.0251 (8)	0.0014 (7)	0.0018 (7)	0.0022 (7)
C26	0.0351 (11)	0.0319 (10)	0.0354 (10)	0.0029 (8)	0.0040 (8)	0.0098 (8)
C27	0.0283 (9)	0.0333 (10)	0.0223 (8)	−0.0030 (8)	0.0058 (7)	−0.0015 (7)
C28	0.0349 (10)	0.0461 (11)	0.0152 (8)	−0.0134 (9)	−0.0041 (7)	0.0043 (8)
C29	0.0343 (11)	0.0661 (14)	0.0238 (9)	−0.0162 (10)	0.0025 (8)	0.0001 (9)
C30	0.0406 (13)	0.106 (2)	0.0276 (11)	−0.0251 (14)	0.0062 (9)	−0.0057 (12)
C31	0.0600 (16)	0.104 (2)	0.0269 (11)	−0.0535 (16)	−0.0015 (11)	0.0117 (13)
C32	0.0750 (18)	0.0756 (17)	0.0241 (11)	−0.0432 (15)	−0.0077 (11)	0.0115 (11)
C33	0.0558 (13)	0.0506 (13)	0.0231 (9)	−0.0228 (11)	−0.0085 (9)	0.0097 (9)
C34	0.0227 (9)	0.0278 (9)	0.0231 (8)	−0.0003 (7)	−0.0023 (7)	0.0006 (7)
C35	0.0266 (10)	0.0352 (10)	0.0395 (11)	−0.0029 (8)	0.0058 (8)	−0.0015 (8)
C36	0.0313 (11)	0.0396 (12)	0.0517 (12)	−0.0095 (9)	0.0025 (9)	0.0079 (10)
C37	0.0468 (13)	0.0277 (10)	0.0486 (12)	−0.0076 (9)	−0.0069 (10)	−0.0009 (9)
C38	0.0498 (13)	0.0300 (10)	0.0338 (10)	0.0017 (9)	−0.0024 (9)	−0.0047 (8)
C39	0.0324 (10)	0.0298 (9)	0.0247 (9)	0.0008 (8)	−0.0001 (7)	−0.0001 (7)

### 1',4'-Diphenyl-1a,1',4',4'',5'',9b-hexahydro-2''H-dispiro[cyclopropa[l]phenanthrene-1,2'-[1,4]epoxynaphthalene-3',3''-thiophene] Geometric parameters (Å, º)

**Table:**

S1—C26	1.799 (2)	C19—H19	0.9500
S1—C27	1.7968 (18)	C19—C20	1.375 (3)
O1—C16	1.443 (2)	C20—H20	0.9500
O1—C23	1.4451 (19)	C20—C21	1.390 (3)
C1—H1	1.0000	C21—H21	0.9500
C1—C2	1.481 (2)	C21—C22	1.386 (3)
C1—C14	1.516 (2)	C22—C23	1.520 (2)
C1—C15	1.525 (2)	C23—C24	1.607 (2)
C2—C3	1.389 (3)	C23—C34	1.503 (2)
C2—C7	1.407 (2)	C24—C25	1.544 (2)
C3—H3	0.9500	C24—C27	1.542 (2)
C3—C4	1.383 (3)	C25—H25A	0.9900
C4—H4	0.9500	C25—H25B	0.9900
C4—C5	1.387 (3)	C25—C26	1.520 (2)
C5—H5	0.9500	C26—H26A	0.9900
C5—C6	1.375 (3)	C26—H26B	0.9900
C6—H6	0.9500	C27—H27A	0.9900
C6—C7	1.405 (3)	C27—H27B	0.9900
C7—C8	1.477 (3)	C28—C29	1.388 (3)
C8—C9	1.403 (3)	C28—C33	1.398 (3)
C8—C13	1.406 (3)	C29—H29	0.9500
C9—H9	0.9500	C29—C30	1.400 (3)
C9—C10	1.374 (3)	C30—H30	0.9500
C10—H10	0.9500	C30—C31	1.385 (4)
C10—C11	1.380 (3)	C31—H31	0.9500
C11—H11	0.9500	C31—C32	1.363 (4)
C11—C12	1.387 (3)	C32—H32	0.9500
C12—H12	0.9500	C32—C33	1.379 (3)
C12—C13	1.392 (3)	C33—H33	0.9500
C13—C14	1.486 (2)	C34—C35	1.396 (3)
C14—H14	1.0000	C34—C39	1.390 (2)
C14—C15	1.518 (2)	C35—H35	0.9500
C15—C16	1.574 (2)	C35—C36	1.389 (3)
C15—C24	1.558 (2)	C36—H36	0.9500
C16—C17	1.526 (3)	C36—C37	1.382 (3)
C16—C28	1.502 (2)	C37—H37	0.9500
C17—C18	1.385 (3)	C37—C38	1.379 (3)
C17—C22	1.392 (3)	C38—H38	0.9500
C18—H18	0.9500	C38—C39	1.387 (3)
C18—C19	1.390 (3)	C39—H39	0.9500
			
C27—S1—C26	95.84 (8)	C21—C20—H20	119.8
C16—O1—C23	98.48 (12)	C20—C21—H21	120.8
C2—C1—H1	114.5	C22—C21—C20	118.35 (19)
C2—C1—C14	117.89 (14)	C22—C21—H21	120.8
C2—C1—C15	124.25 (14)	C17—C22—C23	105.72 (15)
C14—C1—H1	114.5	C21—C22—C17	121.18 (17)
C14—C1—C15	59.93 (11)	C21—C22—C23	133.08 (17)
C15—C1—H1	114.5	O1—C23—C22	100.71 (13)
C3—C2—C1	118.49 (15)	O1—C23—C24	100.10 (12)
C3—C2—C7	119.86 (16)	O1—C23—C34	110.06 (13)
C7—C2—C1	121.65 (16)	C22—C23—C24	108.03 (13)
C2—C3—H3	119.2	C34—C23—C22	117.18 (14)
C4—C3—C2	121.62 (17)	C34—C23—C24	118.02 (13)
C4—C3—H3	119.2	C15—C24—C23	99.36 (12)
C3—C4—H4	120.6	C25—C24—C15	113.10 (13)
C3—C4—C5	118.83 (19)	C25—C24—C23	109.05 (13)
C5—C4—H4	120.6	C27—C24—C15	115.01 (14)
C4—C5—H5	119.9	C27—C24—C23	112.38 (13)
C6—C5—C4	120.28 (18)	C27—C24—C25	107.73 (13)
C6—C5—H5	119.9	C24—C25—H25A	109.7
C5—C6—H6	119.1	C24—C25—H25B	109.7
C5—C6—C7	121.81 (18)	H25A—C25—H25B	108.2
C7—C6—H6	119.1	C26—C25—C24	109.72 (14)
C2—C7—C8	120.13 (16)	C26—C25—H25A	109.7
C6—C7—C2	117.44 (17)	C26—C25—H25B	109.7
C6—C7—C8	122.23 (16)	S1—C26—H26A	110.6
C9—C8—C7	121.69 (17)	S1—C26—H26B	110.6
C9—C8—C13	117.75 (18)	C25—C26—S1	105.53 (13)
C13—C8—C7	120.32 (15)	C25—C26—H26A	110.6
C8—C9—H9	119.1	C25—C26—H26B	110.6
C10—C9—C8	121.7 (2)	H26A—C26—H26B	108.8
C10—C9—H9	119.1	S1—C27—H27A	109.9
C9—C10—H10	119.8	S1—C27—H27B	109.9
C9—C10—C11	120.3 (2)	C24—C27—S1	109.01 (12)
C11—C10—H10	119.8	C24—C27—H27A	109.9
C10—C11—H11	120.4	C24—C27—H27B	109.9
C10—C11—C12	119.2 (2)	H27A—C27—H27B	108.3
C12—C11—H11	120.4	C29—C28—C16	119.05 (18)
C11—C12—H12	119.4	C29—C28—C33	118.78 (19)
C11—C12—C13	121.1 (2)	C33—C28—C16	121.88 (19)
C13—C12—H12	119.4	C28—C29—H29	120.1
C8—C13—C14	121.69 (16)	C28—C29—C30	119.8 (2)
C12—C13—C8	119.78 (17)	C30—C29—H29	120.1
C12—C13—C14	118.43 (17)	C29—C30—H30	120.0
C1—C14—H14	113.6	C31—C30—C29	119.9 (3)
C1—C14—C15	60.32 (11)	C31—C30—H30	120.0
C13—C14—C1	117.45 (15)	C30—C31—H31	119.8
C13—C14—H14	113.6	C32—C31—C30	120.4 (2)
C13—C14—C15	127.23 (15)	C32—C31—H31	119.8
C15—C14—H14	113.6	C31—C32—H32	119.9
C1—C15—C16	115.97 (13)	C31—C32—C33	120.2 (2)
C1—C15—C24	128.04 (14)	C33—C32—H32	119.9
C14—C15—C1	59.75 (11)	C28—C33—H33	119.6
C14—C15—C16	114.74 (14)	C32—C33—C28	120.8 (2)
C14—C15—C24	130.69 (14)	C32—C33—H33	119.6
C24—C15—C16	103.40 (13)	C35—C34—C23	121.14 (16)
O1—C16—C15	100.69 (12)	C39—C34—C23	120.17 (15)
O1—C16—C17	101.03 (13)	C39—C34—C35	118.68 (17)
O1—C16—C28	110.07 (14)	C34—C35—H35	119.8
C17—C16—C15	105.41 (13)	C36—C35—C34	120.43 (18)
C28—C16—C15	115.96 (14)	C36—C35—H35	119.8
C28—C16—C17	120.88 (15)	C35—C36—H36	119.9
C18—C17—C16	135.31 (18)	C37—C36—C35	120.22 (19)
C18—C17—C22	120.09 (18)	C37—C36—H36	119.9
C22—C17—C16	104.55 (15)	C36—C37—H37	120.2
C17—C18—H18	120.8	C38—C37—C36	119.67 (19)
C17—C18—C19	118.5 (2)	C38—C37—H37	120.2
C19—C18—H18	120.8	C37—C38—H38	119.8
C18—C19—H19	119.3	C37—C38—C39	120.47 (19)
C20—C19—C18	121.4 (2)	C39—C38—H38	119.8
C20—C19—H19	119.3	C34—C39—H39	119.7
C19—C20—H20	119.8	C38—C39—C34	120.51 (18)
C19—C20—C21	120.4 (2)	C38—C39—H39	119.7
			
O1—C16—C17—C18	150.6 (2)	C15—C16—C28—C29	−88.3 (2)
O1—C16—C17—C22	−32.00 (15)	C15—C16—C28—C33	85.5 (2)
O1—C16—C28—C29	25.1 (2)	C15—C24—C25—C26	−162.68 (14)
O1—C16—C28—C33	−161.04 (16)	C15—C24—C27—S1	143.94 (12)
O1—C23—C24—C15	−37.87 (14)	C16—O1—C23—C22	−50.74 (14)
O1—C23—C24—C25	80.66 (15)	C16—O1—C23—C24	59.98 (13)
O1—C23—C24—C27	−159.96 (13)	C16—O1—C23—C34	−175.06 (13)
O1—C23—C34—C35	173.15 (15)	C16—C15—C24—C23	3.87 (15)
O1—C23—C34—C39	−8.2 (2)	C16—C15—C24—C25	−111.59 (15)
C1—C2—C3—C4	177.54 (17)	C16—C15—C24—C27	124.06 (15)
C1—C2—C7—C6	−175.70 (16)	C16—C17—C18—C19	177.38 (19)
C1—C2—C7—C8	9.3 (3)	C16—C17—C22—C21	−178.63 (16)
C1—C14—C15—C16	106.82 (15)	C16—C17—C22—C23	0.32 (17)
C1—C14—C15—C24	−116.06 (19)	C16—C28—C29—C30	173.38 (17)
C1—C15—C16—O1	−113.93 (15)	C16—C28—C33—C32	−173.09 (17)
C1—C15—C16—C17	141.36 (15)	C17—C16—C28—C29	142.26 (17)
C1—C15—C16—C28	4.8 (2)	C17—C16—C28—C33	−43.9 (2)
C1—C15—C24—C23	143.16 (16)	C17—C18—C19—C20	0.1 (3)
C1—C15—C24—C25	27.7 (2)	C17—C22—C23—O1	31.36 (16)
C1—C15—C24—C27	−96.66 (19)	C17—C22—C23—C24	−73.07 (16)
C2—C1—C14—C13	−3.8 (2)	C17—C22—C23—C34	150.67 (14)
C2—C1—C14—C15	115.42 (16)	C18—C17—C22—C21	−0.7 (3)
C2—C1—C15—C14	−105.06 (18)	C18—C17—C22—C23	178.22 (15)
C2—C1—C15—C16	150.18 (16)	C18—C19—C20—C21	0.0 (3)
C2—C1—C15—C24	15.1 (3)	C19—C20—C21—C22	−0.4 (3)
C2—C3—C4—C5	−0.6 (3)	C20—C21—C22—C17	0.8 (3)
C2—C7—C8—C9	169.39 (19)	C20—C21—C22—C23	−177.82 (18)
C2—C7—C8—C13	−4.8 (3)	C21—C22—C23—O1	−149.87 (18)
C3—C2—C7—C6	4.5 (3)	C21—C22—C23—C24	105.7 (2)
C3—C2—C7—C8	−170.51 (16)	C21—C22—C23—C34	−30.6 (3)
C3—C4—C5—C6	1.8 (3)	C22—C17—C18—C19	0.3 (3)
C4—C5—C6—C7	0.2 (3)	C22—C23—C24—C15	66.99 (15)
C5—C6—C7—C2	−3.3 (3)	C22—C23—C24—C25	−174.48 (13)
C5—C6—C7—C8	171.56 (18)	C22—C23—C24—C27	−55.10 (17)
C6—C7—C8—C9	−5.4 (3)	C22—C23—C34—C35	59.0 (2)
C6—C7—C8—C13	−179.58 (17)	C22—C23—C34—C39	−122.44 (17)
C7—C2—C3—C4	−2.7 (3)	C23—O1—C16—C15	−57.04 (14)
C7—C8—C9—C10	−173.1 (2)	C23—O1—C16—C17	51.15 (14)
C7—C8—C13—C12	172.01 (17)	C23—O1—C16—C28	−179.94 (13)
C7—C8—C13—C14	−4.2 (3)	C23—C24—C25—C26	87.78 (16)
C8—C9—C10—C11	0.4 (4)	C23—C24—C27—S1	−103.34 (14)
C8—C13—C14—C1	8.3 (2)	C23—C34—C35—C36	177.19 (17)
C8—C13—C14—C15	−63.9 (2)	C23—C34—C39—C38	−178.31 (16)
C9—C8—C13—C12	−2.4 (3)	C24—C15—C16—O1	31.22 (15)
C9—C8—C13—C14	−178.58 (18)	C24—C15—C16—C17	−73.49 (15)
C9—C10—C11—C12	−0.9 (4)	C24—C15—C16—C28	149.93 (15)
C10—C11—C12—C13	−0.3 (3)	C24—C23—C34—C35	−72.9 (2)
C11—C12—C13—C8	2.0 (3)	C24—C23—C34—C39	105.69 (18)
C11—C12—C13—C14	178.27 (19)	C24—C25—C26—S1	36.09 (17)
C12—C13—C14—C1	−167.93 (16)	C25—C24—C27—S1	16.81 (16)
C12—C13—C14—C15	119.9 (2)	C26—S1—C27—C24	3.34 (14)
C13—C8—C9—C10	1.3 (3)	C27—S1—C26—C25	−22.57 (14)
C13—C14—C15—C1	103.49 (19)	C27—C24—C25—C26	−34.44 (18)
C13—C14—C15—C16	−149.69 (17)	C28—C16—C17—C18	29.0 (3)
C13—C14—C15—C24	−12.6 (3)	C28—C16—C17—C22	−153.60 (15)
C14—C1—C2—C3	174.99 (15)	C28—C29—C30—C31	−0.3 (3)
C14—C1—C2—C7	−4.8 (2)	C29—C28—C33—C32	0.7 (3)
C14—C1—C15—C16	−104.76 (16)	C29—C30—C31—C32	1.2 (3)
C14—C1—C15—C24	120.13 (18)	C30—C31—C32—C33	−1.1 (3)
C14—C15—C16—O1	179.19 (13)	C31—C32—C33—C28	0.1 (3)
C14—C15—C16—C17	74.48 (17)	C33—C28—C29—C30	−0.6 (3)
C14—C15—C16—C28	−62.1 (2)	C34—C23—C24—C15	−157.17 (14)
C14—C15—C24—C23	−136.68 (17)	C34—C23—C24—C25	−38.64 (19)
C14—C15—C24—C25	107.85 (19)	C34—C23—C24—C27	80.74 (18)
C14—C15—C24—C27	−16.5 (2)	C34—C35—C36—C37	1.4 (3)
C15—C1—C2—C3	−114.01 (19)	C35—C34—C39—C38	0.3 (3)
C15—C1—C2—C7	66.2 (2)	C35—C36—C37—C38	−0.3 (3)
C15—C1—C14—C13	−119.26 (17)	C36—C37—C38—C39	−0.8 (3)
C15—C16—C17—C18	−105.0 (2)	C37—C38—C39—C34	0.8 (3)
C15—C16—C17—C22	72.46 (16)	C39—C34—C35—C36	−1.4 (3)
